# Effects of ambient temperature and available sugar on bacterial community of *Pennisetum sinese* leaf: An *in vitro* study

**DOI:** 10.3389/fmicb.2022.1072666

**Published:** 2023-01-06

**Authors:** Guangrou Lu, Xiaokang Huang, Lin Li, Chao Chen, Ping Li

**Affiliations:** ^1^College of Animal Science, Guizhou University, Guiyang, China; ^2^Key Laboratory of Animal Genetics, Breeding and Reproduction in the Plateau Mountainous Region, Ministry of Education, Guizhou University, Guiyang, Guizhou, China

**Keywords:** incubation temperature, soluble sugars, *Pennisetum sinese*, bacterial community, alpha diversity

## Abstract

The present *in vitro* study investigated the effects of temperature and available sugar on the bacterial community of *Pennisetum sinese* leaf during fermentation. *P. sinese* leaves were cultured in MRS broth containing 0.4 and 1.6 g sugar and incubated at 25°C and 45°C for 9, 18, and 36 h. The results showed that the dominant phyla during sugar fermentation were Firmicutes, followed by Proteobacteria and Bacteroidetes. Compared to a low incubation temperature (25°C), a high incubation temperature (45°C) decreased the relative abundances of Exiguobacterium and Acinetobacter and increased those of Bacillus and Paenibacillus. Leaf samples incubated at 25°C showed higher bacterial alpha diversity indices than those incubated at 45°C. Principal coordinate analysis revealed that the bacterial community structure was altered by the high incubation temperature. Sugar concentration of 1.6 g/50 ml increased the relative abundances of *Bacillus* and *Klebsiella* but decreased those of *Paenibacillus* and *Serratia* as compared to sugar concentration of 0.4 g/50 ml. pH was the primary factor that influenced the succession of bacterial communities during sugar fermentation in *P. sinese* leaves. In conclusion, ambient temperatures (25°C and 45°C) and high sugar concentration restructured the bacterial communities on *P. sinese* leaves by facilitating the dominance of *Bacillus* and *Paenibacillus*. This study provided insights into the mechanisms by which bacterial communities on *P. sinese* leaves are enriched.

## 1. Introduction

Silage is a high-moisture (65–75%) feed obtained by chopping and fermenting green fodder under anaerobic conditions by using lactic acid bacteria (LAB) to inhibit the growth of various undesirable bacteria. Silage has good palatability and is rich in nutrients, and it is an excellent source of feed for the long-term preservation of livestock.

Silage is ensiled through anaerobic fermentation with epiphytic or inoculated LAB that convert water-soluble carbohydrates (WSC) into organic acids, mainly lactic acid (LA), and thus decrease the pH of silage and inhibits the growth of undesirable microorganisms. The degree of fermentation of silage highly depends on the epiphytic microflora of the ensiled forage material (Eikmeyer et al., 2013; Muck, 2013).

The natural microflora of forage crops participates in silage fermentation. Ohshima et al. (1997) found that adding the fermented juice of epiphytic LAB (FJLB) to silage effectively improved its fermentative quality. Gulfam et al. (2017) reported that the commercially available LAB additive (*Lactiplantibacillus plantarum* MTD-1) did not improve silage quality at 50°C; however, at this high temperature, the natural strains of epiphytic LAB (*Pediococcus acidilactici* GG13 and *Lacticaseibacillus rhamnosus* GG26) improved silage quality by increasing LA content and reducing silage pH and content of butyric acid and ammonia nitrogen (ammonia-N). Nazar et al. (2022) showed that the native and exogenous epiphytic microbiota exhibited strong adaptability and effectively improved the fermentation quality and microbial succession of red clover silage. According to Chavira (2016), silage is produced by preserving plant/crop resources through anaerobic fermentation usually by epiphytic bacteria that convert soluble carbohydrates mainly to LA and small amounts of volatile fatty acids. This definition implies that silage quality may depend on the types of microorganisms present on the leaf surface. Therefore, it is crucial to study the microorganisms that adhere to the leaf surface to enhance silage quality.

Previous studies have revealed the composition of microbial communities attached to the leaf periphery through individual cultures. Ruschmann and Meyer (1934) reported that the acidification rate during silage fermentation depends on epiphytic bacteria growing on forages. Pahlow et al. (2003) showed that the microbial populations of standing or freshly harvested feed crops differ extensively from those found in the silage fermentation process or the final product. Most previous results were obtained under different environmental conditions; however, it remains unclear how temperature and sugar concentration affect the microbial communities attached to the leaf periphery and eventually influence the fermentation quality of silage.

Silage production is a complex process involving diverse microbial flora such as LAB, spoilage-causing bacteria, yeasts, molds, and *Bacillus* (Kung et al., 1987). Both temperature and soluble sugar concentration can affect the composition of microbial communities, which can directly affect silage quality (Zhang et al., 2021) and further affect the rumen microbiota of ruminants (Rajabi et al., 2017). Temperature largely affects the enrichment of bacterial communities on leaves during fermentation. In the fermentation process, maintaining the appropriate temperature is essential to facilitate efficient bacterial growth and metabolite production. Seale et al. (1986) showed that soluble sugar is a limiting factor for obtaining high-quality fermentation products; this implies that soluble sugar is another key factor that influences fermentation quality.

Understanding the epiphytic and inherent microorganisms of forage leaves during *in vitro* fermentation is an interesting and critical issue for producing high-quality silage. Therefore, the present study investigated the bacterial communities present on the surface of *Pennisetum sinese* (king grass) leaves under different incubation temperatures and soluble sugar concentrations. We hypothesized that sugar concentration and temperature might affect the abundance of bacterial communities on the leaf surface of *P. sinese*.

## 2. Materials and methods

### 2.1. Experimental materials

The experimental material *P. sinese* was cut from Guanling County, Anshun City, Guizhou Province (25.94° N, 105.61° E) on July 28, 2021. A bacterial culture medium was used for culturing the bacterial species from *P. sinese* leaves, and the temperature gradient and incubation temperature were controlled by a constant temperature incubator.

### 2.2. Experimental method

The harvested *P. sinese* leaves were brought to the laboratory under aseptic conditions at a low temperature. The samples were prepared on an ultra-clean bench and the relevant materials and instruments required for the experiments were sterilized. The collected *P. sinese* leaves were cut into 135 square pieces of size 1 × 1 cm. The bacterial culture medium was prepared as follows: nutrient broth CM 124 powder (18.0 g) was added to 1 l distilled water, and the mixture was heated and boiled until the powder completely dissolved. The prepared broth was then distributed in separate flasks and autoclaved at 121°C for 15 min. Five square leaflets were taken and cultured in the prepared liquid medium with the following soluble sugar concentration: S1: 0.4 g/50 ml and S2: 1.6 g/50 ml. Three biological replicates were used for each concentration gradient. The cultures were subjected to shaking conditions at 25°C and 45°C for 9, 18, and 36 h. Sample aliquots were taken from the culture medium at the three time points to determine pH, concentrations of ammonia-N and soluble sugar, abundance of microbial communities, and other indicators. Three replicates were used for each treatment.

### 2.3. Chemical index determination

The pH of the fermented medium was measured using a pH meter. Broderick and Kang (1980) method was used to determine ammonia-N in the medium. WSC content was determined by the method of McDonald et al. (1991).

### 2.4. Microbial community analysis

The DNA was extracted with the TGuide S96 Magnetic Soil /Stool DNA Kit (Tiangen Biotech (Beijing) Co., Ltd.) according to manufacturer instructions. The DNA concentration of the samples was measured with the Qubit dsDNA HS Assay Kit and Qubit 4.0 Fluorometer (Invitrogen, Thermo Fisher Scientific, Oregon, United States). The total of PCR amplicons was purified with Agencourt AMPure XP Beads (Beckman Coulter, Indianapolis, IN) and quantified using the Qubit dsDNA HS Assay Kit and Qubit 4.0 Fluorometer (Invitrogen, Thermo Fisher Scientific, Oregon, United States). After the individual quantification step, amplicons were pooled in equal amounts. For the constructed library, use Illumina novaseq 6,000 (Illumina, Santiago CA, United States) for sequencing. The original sequence was processed by BMK Cloud (Biomarker Technologies Co., Ltd., Beijing, China) and was used as a marker for SSU rRNA classification with the SILVA database. The phylogenetic relationship among the bacterial communities was analyzed, and the determination of bacterial alpha diversity, construction of principal coordinate analysis (PCoA) map, and functional prediction of the bacterial communities were then performed using the QIIME2.^1^

### 2.5. Statistical analysis

SAS program version 9.1 (SAS Institute, Cary, NC) was used to compare the changes in chemical composition, microbial population, and bacterial community index during incubation by the Duncan test. Differences were considered statistically significant at *p* < 0.05. We also analyzed Spearman’s correlation between the bacterial community composition and functional prediction and silage parameters at the genus level.

## 3. Results and discussion

### 3.1. Alpha diversity of bacterial community

Table 1 shows the alpha diversity of bacterial communities detected on *P. sinese* leaves. The read values of the samples ranged from 637 to 951, and the Good’s coverage index of most samples was >0.998, thus indicating that most bacteria were detected by high-throughput sequencing technology. At the same soluble sugar concentration, the Chao1 index, abundance-based coverage estimator (ACE) index, Shannon index, and Simpson index of the samples incubated at 25°C were higher than those of the samples incubated at 45°C. This implies that the bacterial community richness and diversity were generally higher at 25°C than at 45°C. This finding can be attributed to bacterial growth inhibition at high temperatures. Previous studies have also reported that high temperatures inhibit bacterial growth. Weinberg et al. (2001) demonstrated that moderate temperatures of 20°C to 30°C are generally preferred for silage fermentation. Zhang et al. (2018) showed that the relative abundance of bacteria in alfalfa silage decreased with the increasing ambient temperature from 20°C to 40°C. In the present study, at the same temperature, the alpha diversity of the bacterial community increased with the increasing sugar concentration (Chao1, ACE, Shannon, and Simpson indices were higher at 1.6-g sugar concentration than at 0.4-g sugar concentration). Similar to the findings of Zi et al. (2022), soluble sugar treatment was found to improve microbial diversity. Soluble sugars can serve as a nutrient for microbial growth. Zi et al. (2022) showed that soluble sugars provided more nutrients for the growth of LAB and promoted LA fermentation to obtain well-preserved silage. In the present study, the increase in alpha diversity was associated with high sugar content. In conclusion, temperature and sugar content altered the alpha diversity of bacterial communities on *P. sinese* leaves.

**Table 1:** Alpha diversity of bacterial communities around leaves.

Groups	OTU number	Coverage	Chao1 index	Ace index	Shannon index	Simpson index
T1S1D9	951	0.999	412.68	383.35	3.24	0.78
T1S1D18	938	0.999	398.02	400.48	2.47	0.69
T1S1D36	640	0.999	282.37	373.98	2.42	0.68
T1S2D9	737	0.998	304.49	311.17	2.51	0.72
T1S2D18	708	0.998	320.44	323.19	2.47	0.69
T1S2D36	671	0.999	330.03	360.57	2.72	0.77
T2S1D9	717	0.999	299.52	298.52	0.91	0.24
T2S1D18	785	0.998	359.05	345.59	1.71	0.54
T2S1D36	809	0.999	363.5	366.13	1.96	0.55
T2S2D9	724	0.998	320.32	323.91	1.27	0.41
T2S2D18	637	0.999	294.18	298.72	1.64	0.54
T2S2D36	816	0.998	369.75	367.77	1.87	0.5

Incubation temperature T1:25°C; T2: 45°C respectively; Soluble sugar concentration S1:0.4 g: S2: 1.6 g; Incubation time D9:9h; D18:18 h: D36: 36 h

### 3.2. Bacterial community composition

**Figure. 1:**
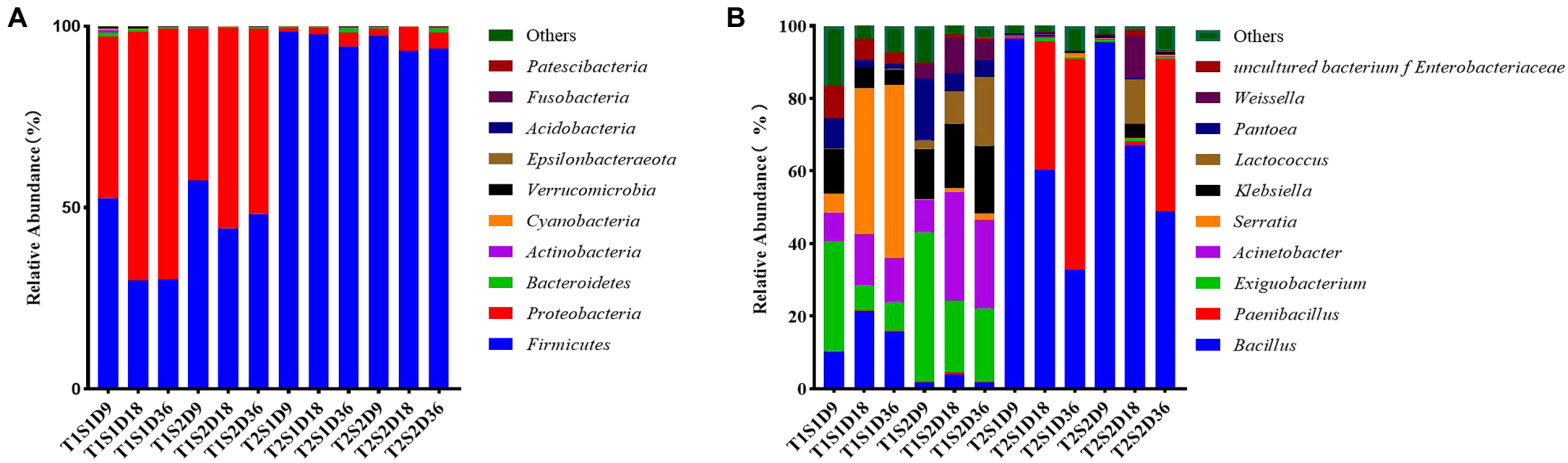
Relative abundances of species *Pennisetum sinese* at the phylum level **(A)** and at the genus level **(B)** after fermentation of at different temperatures and sugar concentrations. T1, 25°C; T2, 45°C; S1, 0.4 g; S2, 1.6 g; D9, 9 h; D18, 18 h; D36, 36 h.

Figure 1A shows the relative abundances of bacterial communities in *P. sinese* silage at the phylum level. In this study, Firmicutes and Proteobacteria were the dominant phyla in the fermentation broth of *P. sinese*; this result was consistent with the findings of Zhang et al. (2021) and Kung et al. (1987) who reported that the bacterial species in the fermentation broth mainly belonged to Firmicutes and Proteobacteria. Long et al. showed similar results for *P. sinese* silage. At the culture temperature T1 (25°C) and soluble sugar content S1 (0.4 g) and S2 (1.6 g), each incubation time period (9 h, 18 h, 36 h) the main bacterial phyla in the fermentation broth were Firmicutes and Proteobacteria. With the increase in culture time, the relative abundance of Firmicutes gradually decreased, reduced by 22.33%, and that of Proteobacteria gradually increased, increasing by 24.13%. At culture temperature T2 (45°C) and soluble sugar content S1 (0.4 g) and S2 (1.6 g), the dominant phylum in the fermentation broth was Firmicutes. At the fermentation temperature of 45°C, the sugar content of 0.4 g, and cultivation time of 9 h, the relative abundance of Firmicutes reached the maximum value of 98.48%. This finding indicated that the culture temperature of 45°C was favorable for the growth of Firmicutes. The high temperature increased the relative abundance of Firmicutes and reduced the proportion of Proteobacteria in the fermentation broth. This finding was similar to the results of another study wherein Firmicutes and Proteobacteria were found to be the most abundant phyla in silage used for hydrolysis and acidification, and their abundance increased by >90% after fermentation (Wang et al., 2019a,b,c). According to Madigan et al. (2017), Proteobacteria are gram-negative bacteria and include pathogenic bacteria such as *Escherichia coli* and *Salmonella*, which compete with LAB to utilize WSCs, thereby decreasing CP content and increasing ammonia-N content. Therefore, the presence of a large number of Firmicutes bacteria may inhibit the growth of LAB.

As shown in Figure 1B, at the incubation temperature T1 (25°C) and soluble sugar content S1 (0.4 g), the dominant bacteria at the genus level were *Serratia*, *Bacillus*, *Acinetobacter*, and *Exiguobacterium* with the relative abundances of 47.58, 21.46, 14.11, and 8.03%, respectively. At the soluble sugar content S2 (1.6 g), the dominant bacterial genus was *Exiguobacterium* with the relative abundance of 41%. Compared to the soluble sugar content of 0.4 g, the relative abundance of *Bacillus* decreased significantly by 19.69% and that of *Serratia* decreased by 4.94% at the soluble sugar content of 1.6 g; this might be due to the inhibition of growth of these bacterial species by the addition of other bacterial species. Cai et al. (1998) reported that LAB inhibit the proliferation of these bacterial species; this occurs mainly because LA fermentation reduces the pH of the medium and inhibits the growth of other bacterial species. Notably, after the temperature was controlled, the abundance of LAB increased when the sugar concentration was increased. This is due to the increase in sugar concentration provided more nutrients, leading to increased microbial proliferation, including that of LAB. Chen and Sun (2011), however, showed that when the proportion of bacteria such as *Serratia* and *Bacillus* is large, the additive effect is not achieved. This finding also explains why previously fermented juice (PFJ) cannot be promoted as a silage additive; this is because the use of PFJ is nonbeneficial mainly due to the low proportion of LAB related to silage and more abundance of other bacteria in PFJ.

At the soluble sugar content of 0.4 g, the relative abundances of *Bacillus* and *Paenibacillus* in the fermentation broth increased by 38.77 and 35.50%, respectively, with the increase of culture temperature; *Exiguobacterium, Acinetobacter*, *Serratia*, and *Klebsiella*, the relative abundances of bacteria decreased by 6.13, 13.70, 40.05, and 5.22%, respectively. At the soluble sugar content of 1.6 g, the relative abundances of *Bacillus* and *Paenibacillus* in the fermentation broth increased with the increase of culture temperature (63.16 and 41.96%, respectively), while the relative abundances of *Exiguobacterium*, *Acinetobacter, Klebsiella, Lactobacillu*s, and *Weissella* decreased by 19.05, 30.06, 13.92, 6.64, and 4.01%, respectively. This finding suggests that when the sugar content is constant, elevated temperature increases the abundance of prophase *Bacillus*, which is gradually replaced by *Paenibacillus*; this might be because of intolerance of *Bacillus* to heat (Murphy et al., 1987). Temperature influences the proliferation of *Lactobacillus*. In the present study, the relative abundance of *Lactobacillus* and *Weissella* decreased with the increase of temperature. Zhang et al. (2018) reported similar findings wherein the relative abundance of LAB in alfalfa silage decreased with the increase of ambient temperature from 20°C to 40°C. The optimal growth temperature of bacteria is approximately 20–30°C, and usually, no bacterial growth occurs at or above 45°C; consequently, bacterial growth is inhibited at high temperatures. This may be because temperature drives enzymatic processes that directly affect bacterial communities such as *Lactobacillus* (Briere et al., 1999). This result also suggests that very high or very low temperature can affect the growth of microbial communities.

The present study also found that increasing the culture temperature and sugar concentration in the green juice fermentation broth did not increase the relative abundance of LAB and did not promote the growth of LAB, but significantly increased the relative abundance of *Bacillus* and *Paenibacillus*. However, in Chen and Sun (2011) the researchers showed that when the proportion of these bacteria such as *Serratia*, *Bacillus*, etc. is large, the addition effect is unstable. In the present study, both ambient temperature and foliar sugar affected the composition of leaf microorganisms, and the microbial composition varied throughout the fermentation process. This explains why the PFJ solution cannot be promoted as a silage additive because its use is unsuitable as it mainly contains a low percentage of LAB associated with silage and a high percentage of other pathogenic bacterial species.

### 3.3. Bacterial community structure

**Figure 2:**
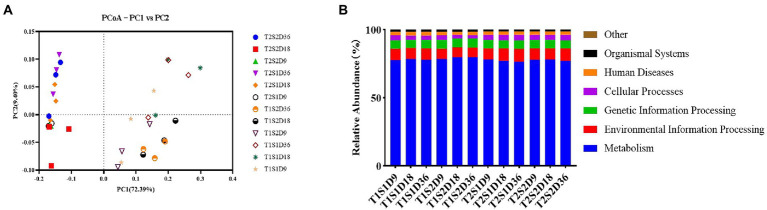
PCoA analysis **(A)** and Functional gene prediction **(B)**.

The PCoA map shows the bacterial community structure of the fermentation broth samples incubated at 25°C and 45°C (Figure 2A). The x-axis and y-axis explained 72.39 and 9.40% of the variance in the bacterial community structure, respectively. Both temperature and sugar concentration promoted changes in bacterial communities, with temperature contributing to the effect of sugar concentration on bacterial growth. According to the functional prediction (Figure 2B), the main functions of bacterial communities in the fermentation broths treated with temperatures T1 and T2 were metabolism, followed by environmental information processing and genetic information processing. This result showed that different temperatures did not influence the metabolic functions of bacteria, thus indicating that bacteria were metabolically active at both temperatures.

### 3.4. Key environmental factors and functional prediction of bacterial community

**Figure 3:**
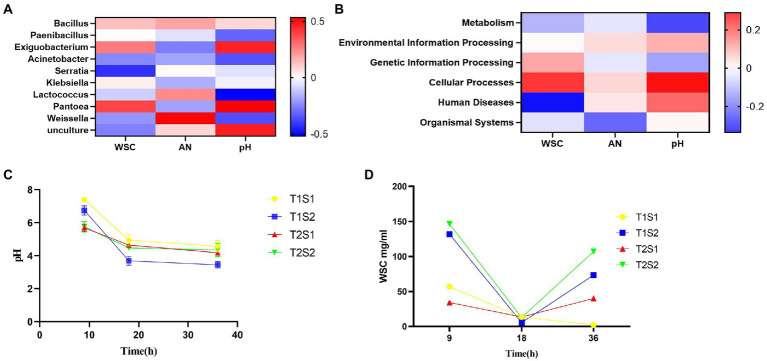
Spearman correlation between WSC, pH and AN in the fermentation broth and the relative abundance of the top 10 bacterial genera **(A)** and functional predictions of bacteria **(B)**; trends in pH **(C)**; and WSC content **(D)** of the fermentation broth with increasing incubation time.

The abundance of specific microorganisms such as LAB in silage can promote its fermentation. Several studies have investigated the correlation between microorganisms and silage fermentation parameters (Li et al., 2019; Ren et al., 2019; Lin et al., 2021). As shown in Figure 3A, *Bacillus, Paenibacillus, Exiguobacterium, Pantoea*, and *Klebsiella* were positively correlated with pH. Xiao and Zhang (2015) reported that soil pH increased with increasing numbers of bacteriophages, and both were approximately highly significant. It is likely that Bacillus has a wide pH range for growth and reproduction, with a general optimum pH of 6.0–9.5, and that Bacillus has limited tolerance to low pH environments: growth is inhibited at pH <4.0. Among these species, *Pantobacillus* showed the strongest correlation value of 0.389. *Serratia*, *Lactococcus*, *Acinetobacter*, *Weissella*, and *Unculture* were negatively correlated with pH, and *Serratia* showed the weakest correlation value of −0.424. The ammonia-N content was positively correlated with *Bacillus*, *Lactococcus*, *Serratia*, *Weissella*, and *Unculture*. *Weissella* showed the strongest correlation value of 0.525, while *Exiguobacterium*, *Paenibacillus*, *Klebsiella*, *Pantoea*, and *Acinetobacter* showed a negative correlation, and the correlation was the weakest (−0.263).

The soluble sugar concentration showed a positive correlation with *Paenibacillus, Bacillus, Exiguobacterium, Klebsiella*, and *Pantoea*, and *Pantoea* showed the strongest correlation value of 0.389. Soluble sugar concentration was negatively correlated with *Serratia, Acinetobacter, Lactococcus, Weissella*, and *Unculture*, and *Serratia* showed the weakest correlation value of −0.424. The relative abundance of *Serratia* was negatively correlated with WSC in the fermentation broth, while *Pantoea* showed a positive correlation with WSC in the fermentation broth. The relative abundances of *Weissella* and *Exiguobacterium* were positively and negatively correlated with ammonia-N in the fermentation broth, respectively. *Lactococcus* was negatively correlated with the pH of the fermentation broth, indicating that lower pH values led to higher relative abundances of *Lactococcus*; this may be due to LA produced by *Lactococcus*, which reduces the pH value of the broth. The pH of the fermentation broth was positively correlated with the relative abundances of *Pantoea* and *Exiguobacterium.*

As shown in Figure 3B, environmental information processing, cellular processes, human diseases, and organic systems showed a positive correlation with pH, and among these, cell transformation showed the strongest correlation value of 0.288. pH was negatively correlated with metabolism and genetic information processing, and the correlation with metabolism was the weakest at −0.251.

Ammonia-N showed a positive correlation with environmental information processing, cellular processes, and human diseases, and the correlation of ammonia-N with human diseases was the strongest (0.050). Metabolism, genetic information processing, and organic systems were negatively correlated with ammonia-N, and metabolism showed the weakest correlation value of −0.206.

Soluble sugar concentration showed a positive correlation with environmental information processing, cellular processes, and genetic information processing, among which cell transformation showed the strongest correlation value of 0.252. Soluble sugar concentration was negatively correlated with metabolism, organic systems, and human diseases, and the correlation with human diseases was the weakest at −0.335. The main function of the bacterial community in the fermentation broth was metabolism, with a relative abundance of 79.98%, followed by environmental information processing and genetic information processing, with a relative abundance of 9.3 and 6.75%, respectively. Metabolism was negatively correlated with WSC, indicating that the higher the bacterial activity, the lower the WSC content.

Silage pH is an important indicator of fermentation quality, and silage with a pH of 4.2 or lower is considered to be well fermented. During the silage production process, beneficial bacteria such as LAB play a decisive role in pH changes; moreover, the temperature will affect the quality of silage by affecting the reproductive and metabolic activities of LAB (Hou et al., 2015). Too low or too high temperature is not conducive to the growth of LAB (Xu et al., 2006). In the present study, pH gradually decreased with the increase of incubation time and temperature (Figure 3C). In general, the pH of the fermentation broth at 45°C was lower than that at 25°C, while the pH at the soluble sugar concentration of 1.6 g was lower than that at 0.4 g, probably due to the promotion of LA production by high sugar concentration, which lowered the pH of the broth. Alli et al. (1984) reported that green fodder with molasses had lower pH, higher acid and acetic acid and soluble sugar content, lower ammoniacal nitrogen content, and better silage quality compared to the control group. Oneil (1986) added 2% of sucrose to alfalfa silage sucrose, pH and ammoniacal nitrogen levels were lower than the control during 2 to 40 days of silage, and soluble sugars in the silage increased. These findings reveal that at a temperature of 45°C and a sugar concentration of 1.6 g, it is easier to reduce the pH value to below 4.2, which is more conducive to silage production and more likely to produce LA. LA in silage is the main product of fermentation and an important indicator to evaluate silage quality. The addition of soluble sugars promotes the reproduction and growth of LAB, resulting in an increased LA content, which decreases pH (Li et al., 2014; Ni et al., 2017; Wang et al., 2019a,b,c).

The WSC content is the limiting factor for silage fermentation, as the minimum WSC content to successfully preserve fresh feed is approximately 3% dry matter (Guyader et al., 2018). As the main effective nutrient for LAB growth, WSC concentration showed an increasing trend with both incubation temperature and sugar concentration; it first decreased and then increased with the increase of incubation time (Figure 3D). Oneil (1986) added 2% of alfalfa silage of sucrose, and the soluble sugars in the silage increased. This is due to LAB used sugar as the substrate during their growth in the early stage, while the growth of LAB was inhibited in the later stage. The presence of a large number of Proteobacteria also inhibits the growth of LAB. Madigan et al. (2017) reported that Proteobacteria are gram-negative bacteria that include pathogenic bacterial species such as *E. coli* and *Salmonella*, which compete with LAB to utilize WSC and thus decrease CP content and increase ammonia-N content. In conclusion, both temperature and sugar content affect pH and WSC. As temperature and sugar content increase, pH decreases and WSC first decreases and then increases, which affects the composition and succession of microorganisms.

## 4. Conclusion

Ambient temperature and available sugar concentration affected the bacterial composition of *P. sinese* leaves. The best fermentation quality was achieved by adding 1.6 g of soluble sugar at 25°C, which increased the relative abundance of LAB and *Weissella*. However, the proportion of *Bacillus* and *Paenibacillus* in the fermentation broth was also found to be relatively large. The use of PFJ may be unsuitable, because it contains a low percentage of LAB associated with silage and a high percentage of other pathogenic anaerobic bacteria. These discoveries suggest that green juice fermentation broth is not suitable as a silage additive.

## Data availability statement

The data presented in the study are deposited in the NCBI repository (https://www.ncbi.nlm.nih.gov/), accession number PRJNA891622.

## Author contributions

GL was mainly responsible for writing the first draft of the article. PL and CC were responsible for revising and annotating the article. GL, XH, and LL participated and completed the experiment. All authors contributed to the article and approved the submitted version.

## Funding

This project was supported by National Key Research & Development Program of China (2021YFD1300302) and Talent Introduction and Scientific Research Program from Guizhou University (GDRHJZ2021-31). We also thank Master Chaosheng Liao Master Xiaolong Tang and Master Maoya Li from Guizhou University for their helps during sampling.

## Conflict of interest

The authors declare that the research was conducted in the absence of any commercial or financial relationships that could be construed as a potential conflict of interest.

## Publisher’s note

All claims expressed in this article are solely those of the authors and do not necessarily represent those of their affiliated organizations, or those of the publisher, the editors and the reviewers. Any product that may be evaluated in this article, or claim that may be made by its manufacturer, is not guaranteed or endorsed by the publisher.

## Footnotes

^1^ https://qiime2.org/

## References

[ref1] Alli I. Fairbairn R. Noroozi E. Baker B. E. (1984). The effects of molasses on the fermentation of chopped whole-plant maize and leucaena. J. Sci. Food Agric. 35, 285–289. doi: 10.1002/jsfa.2740350307

[ref3] Briere J. F. Pracros P. Le Roux A. Y. Pierre J. S. (1999). A novel rate model of temperature-dependent development for arthropods. Environ. Entomol. 28, 22–29. doi: 10.1093/ee/28.1.22

[ref5] Broderick G. A. Kang J. H. (1980). Automated simultaneous determination of ammonia and total amino acids in ruminal fluid and in vitro media. J. Dairy Sci. 63, 64–75. doi: 10.3168/jds.S0022-0302(80)82888-8, PMID: 7372898

[ref6] Cai Y. Benno Y. Ogawa M. Ohmomo S. Kumai S. Nakase T. (1998). Influence of lactobacillus spp. from an inoculant and of Weissella and Leuconostoc spp. from forage crops on silage fermentation. Appl. Environ. Microbiol. 64, 2982–2987. doi: 10.1128/AEM.64.8.2982-2987.1998, PMID: 9687461 PMC106803

[ref7] Chavira J. S. (2016). Potential use of nonconventional silages in ruminant feeding for tropical and subtropical areas. Adv. Silage Product. Utiliz. iz 85–98. doi: 10.5772/64382

[ref01] Chen D. Sun C. (2011). Practical clinical microbiology test and atlas. Beijing: People’s Health Publishing House. 201, 769–775.

[ref9] Eikmeyer F. G. Köfinger P. Poschenel A. Jünemann S. Zakrzewski M. Heinl S.. (2013). Metagenome analyses reveal the influence of the inoculant lactobacillus buchneri CD034 on the microbial community involved in grass ensiling. J. Biotechnol. 167, 334–343. doi: 10.1016/j.jbiotec.2013.07.021, PMID: 23880441

[ref12] Gulfam A. Guo G. Tajebe S. Chen L. Liu Q. Yuan X.. (2017). Characteristics of lactic acid bacteria isolates and their effect on the fermentation quality of Napier grass silage at three high temperatures. J. Sci. Food Agric. 97, 1931–1938. doi: 10.1002/jsfa.7998, PMID: 27539868

[ref13] Guyader J. Baron V. S. Beauchemin K. A. (2018). Corn forage yield and quality for silage in short growing season areas of the Canadian prairies. Agronomy 8:164. doi: 10.3390/agronomy8090164

[ref15] Hou X. Ye L. Wei X. (2015). Screening of lactic acid bacteria for silage and their biological properties. J. Northwest Agric. Forest. Univ. 43, 183–192. doi: 10.13207/j.cnki.jnwafu.2015.01.019

[ref16] Kung L. Satter L. D. Jones B. A. Genin K. W. Sudoma A. L. Enders G. L.. (1987). Microbial inoculation of low moisture alfalfa silage. J. Dairy Sci. 70, 2069–2077. doi: 10.3168/jds.S0022-0302(87)80255-2

[ref18] Li P. Zhang Y. Gou W. Cheng Q. Bai S. Cai Y. (2019). Silage fermentation and bacterial community of bur clover, annual ryegrass and their mixtures prepared with microbial inoculant and chemical additive. Anim. Feed Sci. Technol. 247, 285–293. doi: 10.1016/j.anifeedsci.2018.11.009

[ref19] Li M. Zi X. Zhou H. Hou G. Cai Y. (2014). Effects of sucrose, glucose, molasses and cellulase on fermentation quality and in vitro gas production of king grass silage. Anim. Feed Sci. Technol. 197, 206–212. doi: 10.1016/j.anifeedsci.2014.06.016

[ref20] Lin H. Lin S. Awasthi M. K. Wang Y. Xu P. (2021). Exploring the bacterial community and fermentation characteristics during silage fermentation of abandoned fresh tea leaves. Chemosphere 283:131234. doi: 10.1016/j.chemosphere.2021.131234 34153916

[ref03] Madigan M. T. Bender K. S. Buckley D. H. Sattley W. M. Stahl D. A. (2017). Brock Biology of Microorganisms 15 edn.

[ref04] McDonald P. Henderson A. R. Heron S. J. E. (1991). The biochemistry of silage. Chalcombe publications.

[ref21] Muck R. E. (2013). Recent advances in silage microbiology. Agric. Food Sci. 22, 3–15. doi: 10.23986/afsci.6718

[ref22] Murphy P. A. Dowds B. C. McConnell D. J. Devine K. M. (1987). Oxidative stress and growth temperature in Bacillus subtilis. J. Bacteriol. 169, 5766–5770. doi: 10.1128/jb.169.12.5766-5770.1987, PMID: 3119568 PMC214122

[ref23] Nazar M. Ullah M. W. Wang S. Zhao J. Dong Z. Li J.. (2022). Exploring the epiphytic microbial community structure of forage crops: their adaptation and contribution to the fermentation quality of forage sorghum during ensiling. Bioengineering 9:428. doi: 10.3390/bioengineering9090428, PMID: 36134971 PMC9495736

[ref24] Ni K. Wang F. Zhu B. Yang J. Zhou G. Pan Y. I.. (2017). Effects of lactic acid bacteria and molasses additives on the microbial community and fermentation quality of soybean silage. Bioresour. Technol. 238, 706–715. doi: 10.1016/j.biortech.2017.04.055, PMID: 28501002

[ref26] Ohshima M. Cao L. M. Kimura E. Ohshima Y. Yokota H. (1997). Influence of addition of previously fermented juice to alfalfa [Medicago sativa] ensiled at different moisture contents. J. Japan. Soc. Grassland Sci. 43, 56–58.

[ref27] Oneil K. A. (1986). Effect of microbial inoculants and sucrose on fermentation of alfalfa haylage. J. Anim. Sci. 63, 286–287.

[ref28] Pahlow G. Muck R. E. Driehuis F. Elferink S. J. O. Spoelstra S. F. (2003). Microbiology of ensiling. Silage Sci. Technol. 42, 31–93. doi: 10.2134/agronmonogr42.c2

[ref29] Rajabi R. Tahmasbi R. Dayani O. Khezri A. (2017). Chemical composition of alfalfa silage with waste date and its feeding effect on ruminal fermentation characteristics and microbial protein synthesis in sheep. J. Anim. Physiol. Anim. Nutr. 101, 466–474. doi: 10.1111/jpn.12563, PMID: 27600493

[ref30] Ren F. He R. Zhou X. Gu Q. Xia Z. Liang M.. (2019). Dynamic changes in fermentation profiles and bacterial community composition during sugarcane top silage fermentation: a preliminary study. Bioresour. Technol. 285:121315. doi: 10.1016/j.biortech.2019.121315, PMID: 30965280

[ref02] Ruschmann G. Meyer W. (1934). Das Verhalten der auf grünen Pflanzen vorkommenden Coli-und coliähnlichen Bakterien gegenüber Säuren. Arch. Microbiol. 5, 477–501.

[ref31] Seale D. R. Henderson A. R. Pettersson K. O. Lowe J. F. (1986). The effect of addition of sugar and inoculation with two commercial inoculants on the fermentation of lucerne silage in laboratory silos. Grass Forage Sci. 41, 61–70. doi: 10.1111/j.1365-2494.1986.tb01793.x

[ref32] Wang Y. Chen X. Wang C. He L. Zhou W. Yang F.. (2019c). The bacterial community and fermentation quality of mulberry (Morus alba) leaf silage with or without lactobacillus casei and sucrose. Bioresour. Technol. 293:122059. doi: 10.1016/j.biortech.2019.122059, PMID: 31476563

[ref33] Wang C. He L. Xing Y. Zhou W. Yang F. Chen X.. (2019a). Effects of mixing Neolamarckia cadamba leaves on fermentation quality, microbial community of high moisture alfalfa and stylo silage. Microb. Biotechnol. 12, 869–878. doi: 10.1111/1751-7915.13429, PMID: 31237418 PMC6680604

[ref34] Wang C. He L. Xing Y. Zhou W. Yang F. Chen X.. (2019b). Fermentation quality and microbial community of alfalfa and stylo silage mixed with Moringa oleifera leaves. Bioresour. Technol. 284, 240–247. doi: 10.1016/j.biortech.2019.03.129, PMID: 30947138

[ref35] Weinberg Z. G. Szakacs G. Ashbell G. Hen Y. (2001). The effect of temperature on the ensiling process of corn and wheat. J. Appl. Microbiol. 90, 561–566. doi: 10.1046/j.1365-2672.2001.01276.x, PMID: 11309068

[ref36] Xiao J. Zhang M. (2015). Characteristics of regional distribution of soil bacterium and correlation with soil pH in tobacco plantation in Xiangxi Province. Crop. Res. 29, 635–638. doi: 10.3969/j.issn.1001-5280.2015.06.14

[ref37] Xu Q. Zhou He Y. Li S. Han J. Bai C. (2006). Effects of storage period and addition of green juice fermentation broth on bagged alfalfa silage. J. Grassland 14, 129–146.

[ref39] Zhang F. Miao F. Wang X. Lu W. Ma C. (2021). Effects of homo-and hetero-fermentative lactic acid bacteria on the quality and aerobic stability of corn silage. Can. J. Anim. Sci. 101, 761–770. doi: 10.1139/cjas-2019-0170

[ref40] Zhang Q. Yu Z. Wang X. Tian J. (2018). Effects of inoculants and environmental temperature on fermentation quality and bacterial diversity of alfalfa silage. Anim. Sci. J. 89, 1085–1092. doi: 10.1111/asj.12961, PMID: 29845704

[ref42] Zi X. Liu Y. Chen T. Li M. Zhou H. Tang J. (2022). Effects of sucrose, glucose and molasses on fermentation quality and bacterial Community of Stylo Silage. Fermentation 8:191. doi: 10.3390/fermentation8050191

